# Internet-Delivered Cognitive Behavioral Therapy in Patients With Irritable Bowel Syndrome: Systematic Review and Meta-Analysis

**DOI:** 10.2196/35260

**Published:** 2022-06-10

**Authors:** Hyunjung Kim, Younjae Oh, Sun Ju Chang

**Affiliations:** 1 School of Nursing Research Institute of Nursing Science Hallym University Chuncheon Republic of Korea; 2 College of Nursing Research Institute of Nursing Science Seoul National University Seoul Republic of Korea

**Keywords:** cognitive behavioral therapy, irritable bowel syndrome, internet, symptom, quality of life

## Abstract

**Background:**

Irritable bowel syndrome is a common functional gastrointestinal disorder that negatively affects all aspects of life. With the widespread use of the internet, internet-delivered cognitive behavioral therapy has been developed and applied to control symptoms and improve the quality of life of those with irritable bowel syndrome. However, few studies have systematically reviewed the effectiveness of internet-delivered cognitive behavioral therapy on irritable bowel syndrome.

**Objective:**

This study aimed to systematically review studies that examined the use of internet-delivered cognitive behavioral therapy in patients with irritable bowel syndrome and to evaluate the effects of internet-delivered cognitive behavioral therapy on the improvement of symptom severity, quality of life, psychological status, and cost-effectiveness.

**Methods:**

This meta-analysis involved the search of 6 databases for relevant publications. From the 1224 publications identified through database searches, 9 randomized controlled trials were finally included in the analysis.

**Results:**

The internet-delivered cognitive behavioral therapies including exposure-based cognitive behavioral therapy, cognitive behavioral therapy for self-management, and cognitive behavioral therapy for stress management were provided in 5 to 13 sessions for 5 to 10 weeks. Internet-delivered cognitive behavioral therapy had medium-to-large effects on symptom severity (standardized mean difference [SMD] –0.633; 95% CI –0.861 to –0.4304), quality of life (SMD 0.582; 95% CI 0.396-0.769), and cost-effectiveness (–0.372; 95% CI –0.704 to –0.039) at postintervention. The effects on symptom severity remained over time even after the intervention, short-term follow-up (SMD –0.391; 95% CI –0.560 to –0.221), and long-term follow-up (SMD –0.357; 95% CI –0.541 to –0.172). There was no significant difference in psychological status, including anxiety and depression, in those with irritable bowel syndrome compared to the controls during the postintervention period.

**Conclusions:**

This review demonstrates that internet-delivered cognitive behavioral therapy could be a cost-effective intervention for improving symptoms and the quality of life in patients with irritable bowel syndrome. However, studies are still insufficient regarding the use of internet-delivered cognitive behavioral therapy in these patients; therefore, more high-quality studies are required in the future.

## Introduction

Irritable bowel syndrome (IBS), a common chronic gastrointestinal disorder, has a high prevalence of 5% to 20% worldwide [1]. Most patients with IBS experience intestinal symptoms, such as bloating, cramps, diarrhea, and constipation, in addition to abdominal pain and discomfort, for an average of 8.1 days per month [2]. Psychological symptoms include depression, anxiety, sensitivity, anger, and somatization. The symptoms can be so severe that up to 38% of patients consider suicide [3]. IBS is a social problem that causes absence, anxiety about unemployment, decreased work productivity, and increased medical costs, while also being a health problem that causes stress and negatively affects the quality of life (QOL) [4]. Therefore, symptom management and health promotion are essential in patients with IBS.

Although the mechanism has not been identified exactly, IBS can be explained with a biopsychosocial model in which somatization symptoms occur as psychosocial factors influencing the physiological functions of the brain-gut axis [5]. IBS treatment includes providing psychological comfort to the patient and assessing and correcting factors that stimulate bowel movement and sensation. The patients’ quality of life (QOL) can be enhanced by improving their symptoms through lifestyle modification, the use of appropriate medication, and psychiatric treatment [6].

Based on a cognitive-behavioral model in which situation, thoughts, emotions, behaviors, and physiological responses interact with each other, cognitive behavioral therapy (CBT) has been considered as a treatment choice for IBS. CBT is a broad intervention that can include the following features: educational therapy for IBS; cognitive therapy to understand the relationship between thought, emotions, and IBS symptoms; and behavioral therapy, such as stress management, self-management, and self-help treatment [7]. CBT-based exposure therapy, including exposure training to symptom control by exposure to feared and avoided stimuli, has also been used for patients with IBS [8]. CBT is effective in improving the physical and psychological symptoms of IBS and the QOL [9,10]. In a meta-analysis of 18 randomized controlled trials, CBT was found to be more effective in patients with IBS than in control groups consisting of, for instance, those on waiting lists or receiving basic support [7]. With the implementation of CBT, it is expected that patients with IBS will gradually become healthier, more active, and more confident [5]. However, it is difficult for most patients to access CBT due to a shortage of trained therapists, especially in rural areas [11].

As the internet becomes popular worldwide, internet-delivered CBT (ICBT) can compensate for the treatment limitations of CBT. Whereas computerized CBT provides therapy via a computer system but without a therapist’s input, ICBT adds that advantage while keeping the therapist’s contact to a minimum [12]. ICBT consists of online psychoeducational material provided via the internet and therapist guidance, which can include providing feedback or encouragement via SMS text message, email, or chat rooms [13]. It has the advantages of reduced time for the therapist compared to conventional CBT and the ability for patients to access the treatment at any time and place [11]. Accordingly, ICBT has been applied to many psychiatric disorders, and a systematic review showed efficacy in 25 clinical applications, including psychiatric (eg, depression and anxiety), functional (eg, chronic pain and IBS), and eating disorders. Substantial evidence for the positive effects of ICBT on depression, panic disorder, and social phobia can be found [12]. Some randomized controlled trials (RCTs) have recently proven the effects of ICBT on patients with IBS. However, these studies have limitations due to the small sample size and heterogeneity [8,14,15]. To date, only a few papers have systematically reviewed the intervention methods and effectiveness of ICBT in this population. Therefore, this study attempts to comprehensively review and analyze the contents and effects of ICBT programs currently being tested in patients with IBS.

The objectives of this study are to systematically review the studies that examined the application of ICBT in patients with IBS and to evaluate the effects of ICBT on the improvement in symptom severity of IBS, QOL, anxiety, depression, and cost-effectiveness. This will provide comprehensive evidence regarding this topic.

## Methods

### Study Design

This study is a meta-analysis conducted to measure the effect size of ICBT in patients with IBS.

### Literature Search

This study was conducted in accordance with the systematic literature review guidelines suggested by the PRISMA (Preferred Reporting Items for Systematic Reviews and Meta-Analysis) group [16]. A literature search was conducted using the popular search databases, PubMed, Cochrane Library, and PsycINFO, as well as Korean databases, Korean Studies Information Service System (KISS), Korean Medical Database (KMBASE), and Research Information Sharing Service (RISS) up to June 2020. Related studies in the reference list were searched for to find additional studies. Search key terms merged “irritable bowel syndrome” with “cognitive behavior therapy” or “cognitive therapy” or “cognitive psychotherapy.” The complete search strategy is provided in Multimedia Appendix 1. The parameters set for the search were RCTs, journal articles, English or Korean language, and adults. The year of publication was not limited, so we could obtain a comprehensive overview of how ICBT was provided to patients with IBS. To prevent missing relevant publications, the general key term “CBT” (and not “ICBT”) was selected as the key term, and abstracts of studies were screened for eligibility.

### Study Selection

The data inclusion criteria were based on the PICO framework (Participant, Intervention, Comparator, Outcome), where the participant was defined as an adult patient with underlying IBS, intervention consisted of at least one of the elements of CBT and was delivered over the internet, the comparator was a group that did not receive ICBT, and the outcome was the measurable effects of ICBT.

The inclusion criteria were following: an RCT research design, adult participants with IBS; ICBT intervention (exposure-based ICBT, ICBT for self-management, ICBT for stress management), controls (patients on a waiting list who receive intervention after the treatment group, consisting of standard care, psychological treatment, or usual medical treatment), and measurable outcomes (IBS symptom severity, QOL, anxiety, depression, cost-effectiveness, visceral sensitivity, cognitive function, disability, stress, relief).

The exclusion criteria were the following: a non-RCT or secondary data analysis, studies in which ICBT was provided to both the experimental and control groups, studies with an objective other than assessing the effects of ICBT, and studies that presented insufficient data to measure the effect size.

First, duplicates were removed from the list of publications found via database searches. The titles and abstracts of publications were screened, and then the full-text studies were reviewed for eligibility. If the full text was not available, it was requested from the author. If the abstract was insufficient to determine whether the paper met the inclusion criteria, the full text was also searched for and screened. According to the inclusion and exclusion criteria, 2 researchers (HK and YO) reviewed and selected the studies separately. In the case of disagreement between them, a third researcher’s (SC) opinion was to be consulted; however, the study selection results were consistent among the researchers.

### Data Collection and Quality Assessment

Two researchers (HK and YO) independently collected the data from the selected papers using a data extraction form. The form was used to obtain data on the author, year, country, sample characteristics (sample size, mean age), intervention (type, duration, length of follow-up), control category (waiting list, standard care, other psychological therapy), primary and secondary outcome variables, intention to treat (ITT), and results. The primary outcome was the effect of ICBT on IBS symptom severity, which was evaluated using the following: the IBS-Symptom Severity Scale (IBS-SSS) [17], the Gastrointestinal Symptom Rating Scale (GSRS)-IBS [18], and the Bowel Symptom Severity Scale (BSSS) [19]. The secondary outcomes included QOL measured with the IBS-QOL [20], mood status measured with the Hospital Anxiety and Depression Scale (HADS) [21], the State-Trait Anxiety Inventory (STAI-S) [22], the Montgomery Asberg Depression Rating Scale (MADRS) [23], and the Center for Epidemiological Studies Depression scale (CES-D) [24]. Cost-effectiveness was measured using the Trimbos/Institute of Medical Technology Assessment Cost Questionnaire for Psychiatry (TIC-P) [25].

The methodological quality of the selected studies was assessed using the 7 criteria of the Cochrane’s Risk of Bias of the Cochrane Collaboration [26]. Two researchers (HK and YO) independently evaluated the risk of bias in individual papers, and if the results were inconsistent, a consensus was reached through discussion.

### Data Analysis

Comprehensive Meta-Analysis version 3.3 (Biostat) was used to assess heterogeneity and publication bias and to calculate the effect size. Heterogeneity was analyzed using the *Q* test and *I*^2^ test. When the significance level of the *Q* statistic was less than 0.05, the results were considered heterogeneous. The *I^2^* value means that the closer the value is to 100%, the higher the heterogeneity: 25% (small), 50% (medium), and 75% (large) [27]. In this study, a fixed effects model was used when the studies were homogenous, and a random effects model was used when the studies were heterogeneous. For analyzing the effect size in subgroups, the recommended fixed effects model was used [28].

To verify the effect size, standardized mean difference (SMD) values with 95% CIs were calculated because the outcome variables were measured with several tools. The effect size for each outcome was analyzed postintervention. The primary outcome, IBS symptom severity, was further evaluated for the effects of short-term (4 to 6 months from intervention) and long-term (12 to 24 months) follow-up. Additionally, IBS symptom severity was analyzed in subgroups to evaluate the effect size according to the type of intervention, such as self-management and exposure therapy. Cohen’s *d* guidelines were used to interpret the effect size, where a value of 0.2 indicated a small, 0.5 a medium, and 0.8 a large effect size [29]. For the QOL, a positive effect size indicated improvement, while a negative effect size of IBS symptom severity, psychological status, and cost indicated improvement.

The publication bias was assessed using Egger’s regression intercept: if Egger’s regression intercept was not significant, publication bias was considered present. If publication bias was present, the effect size would be corrected using Duval and Tweedie’s trim and fill [30].

## Results

**Figure 1:**
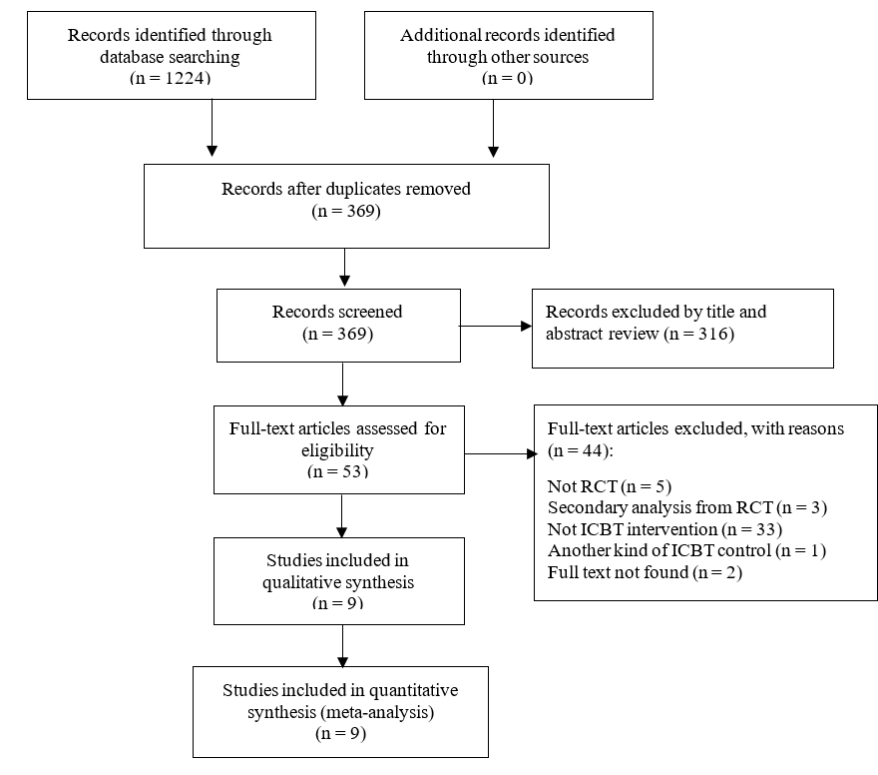
Flow diagram for study inclusion. ICBT: internet-delivered cognitive behavioral therapy; RCT: randomized controlled trial.

The titles and abstracts for 369 publications were screened after 855 duplicates were excluded among the 1224 publications initially identified from the search of 6 databases. Full-text screening of 53 studies was performed for eligibility, but 2 studies without the full text were eventually unable to be accessed due to no response being received from the authors. Finally, 9 studies were selected for the analysis (Figure 1).

### Study Characteristics

Ultimately, 9 RCT studies were included in the analysis (Table 1). A summary of the data extraction results are presented in Multimedia Appendix 2. The studies were published between 2009 and 2019, and 6 out of the 9 studies were conducted by 2 different teams, one led by Everitt [15,31,32] and the other by Ljotsson [8,33,34]. One study was only conducted among women [14], while the rest of the studies included between 73.8% to 84.7% females, with an average age ranging from 18.5 to 44.4 years. All studies excluded participants with medical conditions that could affect the results, such as other gastrointestinal disorders (inflammatory bowel disease, celiac disease, rectal bleeding, and colorectal carcinoma) or psychiatric disorders (severe depressive symptoms, suicide ideation, psychosis, manic episodes, anorexia, and substance dependence). With the exception of 1 study [11] in which participants were included based on the self-report of a diagnosis with IBS by a medical professional, 7 studies included those diagnosed by the Rome III criteria. One study included both patients who self-reported a diagnosis by a medical professional and those who met the Rome III criteria [35]. The mean score of baseline IBS symptom severity ranged from 241 to 265 (out of 500) in 3 studies using IBS-SSS [15,31,32], 42.2 to 53.6 in 4 studies using GSRS [8,11,33,34], and 27.9 in a study using BSSS [14].

**Table 1:** Characteristics of included studies.

Authors	Female (%)	Age (years)	Intervention	Duration	Length of f/u^a^	Controls	ITT^b^
Andersson et al [35]	84.7	34.6	Exposure therapy (n=42)	10 w^c^/5 s^d^	10 w, 3 m^e^, 12 m	Waiting list (n=43)	Yes
Everitt et al [32]	77.8	44.4	Self-management (n=45)	6 w/8 s	6 w, 12 w	Standard care (n=45)	Yes
Everitt et al [15]	76.3	42.9	Self-management (n=185)	9 w/8 s	3 m, 6 m, 12 m	Standard care (n=187)	Yes
Everitt et al [31]	N/A^f^	42.9	Self-management (n=99)	9 w/8 s	24 m	Standard care (n=105)	Yes
Hunt et al [11]	81.5	38.5	Exposure therapy (n=28)	5 w/5 s	5 w, 3 m	Waiting list (n=26)	Yes
Lee et al [14]	100	18.5	Stress management (n=48)	6 w/13 s	2 w, 6 w, 18 w	Waiting list (n=70)	N/A
Ljótsson et al [33]	84.7	34.6	Exposure therapy (n=42)	10 w/5 s	10 w, 3 m	Waiting list (n=43)	Yes
Ljótsson et al [8]	73.8	34.9	Exposure therapy (n=30)	10 w/5 s	10 w, 12 m	Waiting list (n=31)	Yes
Ljótsson et al [34]	79	38.9	Exposure therapy (n=98)	10 w/5 s	10 w, 6 m	Internet-delivered stress management (n=97)	Yes

^a^f/u: follow-up.

^b^ITT: intention to treat.

^c^w: weeks.

^d^s: sessions.

^e^m: month.

^f^N/A: not available

### ICBT Program Characteristics

Among the types of CBT provided through the internet, exposure-based CBT was provided in 5 studies [8,11,33-35], CBT for self-management in 3 studies [15,31,32], and CBT for stress management in 1 study [14]. ICBT was provided as 5 to 13 sessions during a period of 5 to 10 weeks. For the control group, a waiting list was applied in 5 studies [8,11,14,33,35], standard care in 3 studies [15,31,32], and stress management techniques that did not involve CBT were applied in 1 study [34]. In all studies, a therapist contacted the patients in the ICBT group via email, telephone, or internet platform; the main contact method was email in 6 of the 9 studies (67%). The average time of therapist contact was reported in 6 studies (67%) and varied from 73 to 165 minutes in total.

After the intervention, postintervention assessments were performed, and follow-up assessments were performed at 3, 4, 6, 12, and 24 months. However, the follow-up assessments for studies with patients on a waiting list as a control group were only performed in the experimental group. In 1 study [14], ICBT was also administered to the control group (waiting list) after all follow-up assessments were completed.

For the primary outcome, 1 study [35] evaluated cost-effectiveness, while all other studies evaluated the symptom severity of IBS. In addition, QOL, anxiety, depression, visual sensitivity index, adequate relief, and cognitive function were evaluated as outcome variables. ITT data were reported in all except 1 study [14].

### Quality Assessment

**Figure 2:**
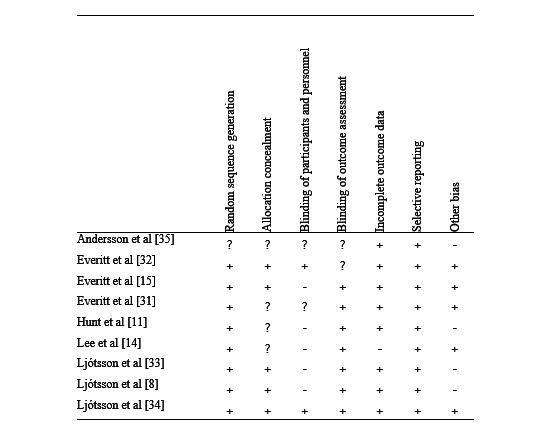
Quality assessment of selected studies.

The methodological quality of the 9 included studies varied (Figure 2): 8 studies (89%) met at least 4 of the quality criteria, including 1 study [34] that met all 7 criteria. Only 1 study (11%) met 2 of the criteria [35]. All studies had a random sequence generation except for 1 study [35], 5 studies provided adequate information on allocation concealment, and only 2 studies [32,34] described the blinding of participants and personnel clearly. All studies involved the blinding of outcome assessments except for 2 studies [32,35], which did not provide sufficient information.

Regarding incomplete outcome data, all studies reported outcome data analysis completely except for 1 [14]. All studies reported all expected outcomes, including those that were prespecified to minimize bias due to selective outcome reporting. Finally, 5 studies appeared to be free of biased sources [14,15,31,32,34], whereas the other 4 studies did not report the outcomes from the waiting list control group in the follow-up stage.

### Effects of ICBT on Patients with IBS

#### Symptom Severity of IBS

IBS symptom severity was the most reported variable as a primary outcome (7 out of 9 studies) [8,11,14,15,32-34]. Since 7 studies showed significant heterogeneity (*I^2^*=56.01; *P*=.03), the overall effect on symptom severity was analyzed using a random model in postintervention. The subgroup analysis was performed using a fixed model.

**Figure 3:**
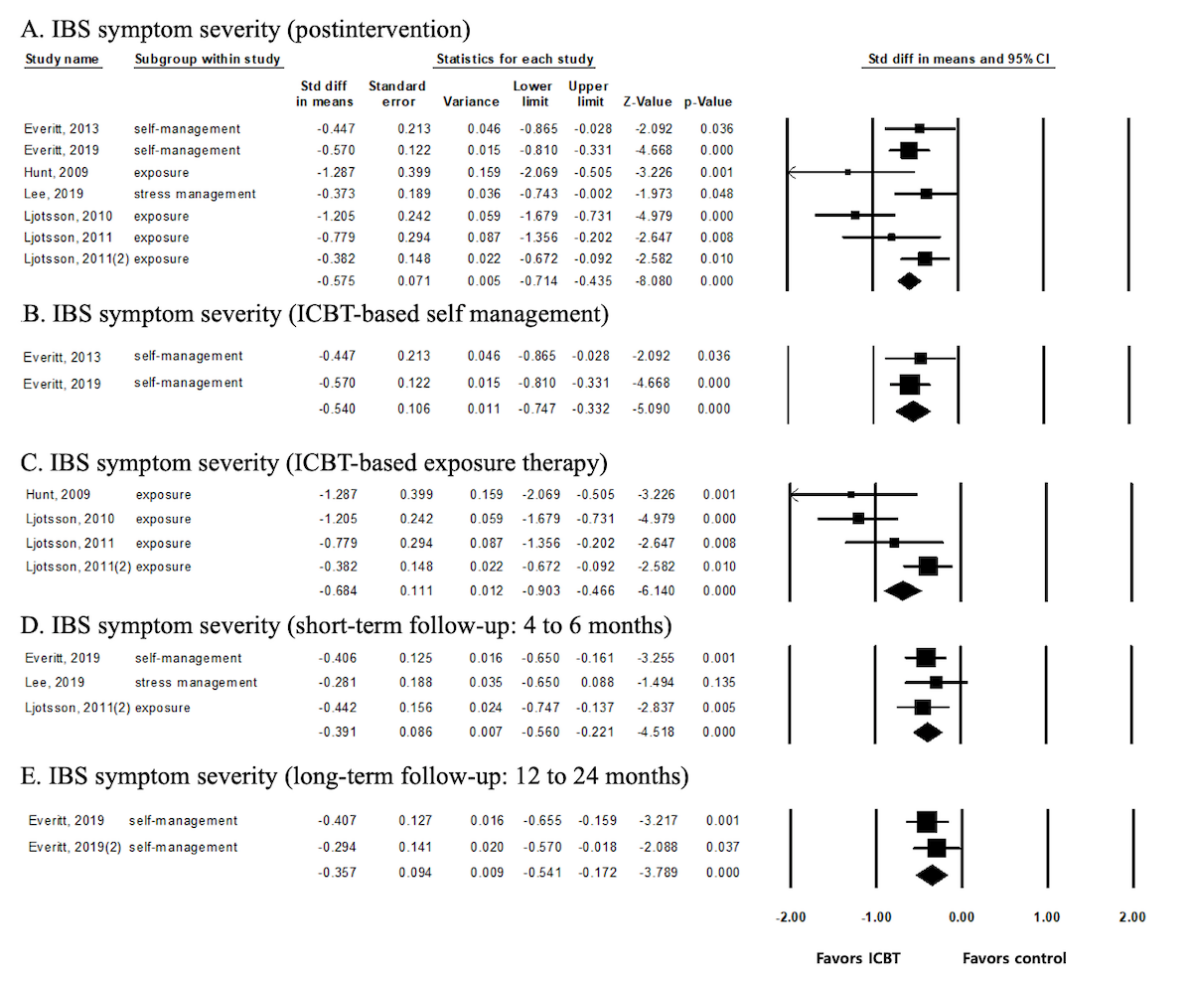
Effects size of ICBT on IBS symptom severity. IBS: irritable bowel syndrome; ICBT: internet-delivered cognitive behavioral therapy; STD: standard.

Postintervention, the ICBT group had a significant reduction in IBS symptom severity compared with the control group (SMD –0.575; 95% CI –0.714 to –0.435), indicating a medium-to-large overall effect size (Figure 3A). In the subgroup analysis, we evaluated whether the effect differed according to the type of intervention. The group receiving ICBT-based self-management intervention reported significantly reduced symptom severity compared with the control group (SMD –0.540; 95% CI –0.747 to –0.332). Additionally, the group that received exposure therapy was compared with the control group, and there was a significant effect on symptom severity (SMD –0.684; 95% CI –0.903 to –0.466; Figure 3B and 3C). ICBT-based stress management was evaluated in 1 study [14], so a subgroup analysis could not be conducted.

Three short-term follow-up studies [14,15,34] had small-to-medium effect sizes in the ICBT group (SMD –0.391; 95% CI –0.560 to –0.221), and the effects remained even in the 2 long-term follow-up studies (SMD –0.357; 95% CI –0.541 to –0.172; Figure 3D and 3E) [15,31].

#### Quality of Life

**Figure 4:**
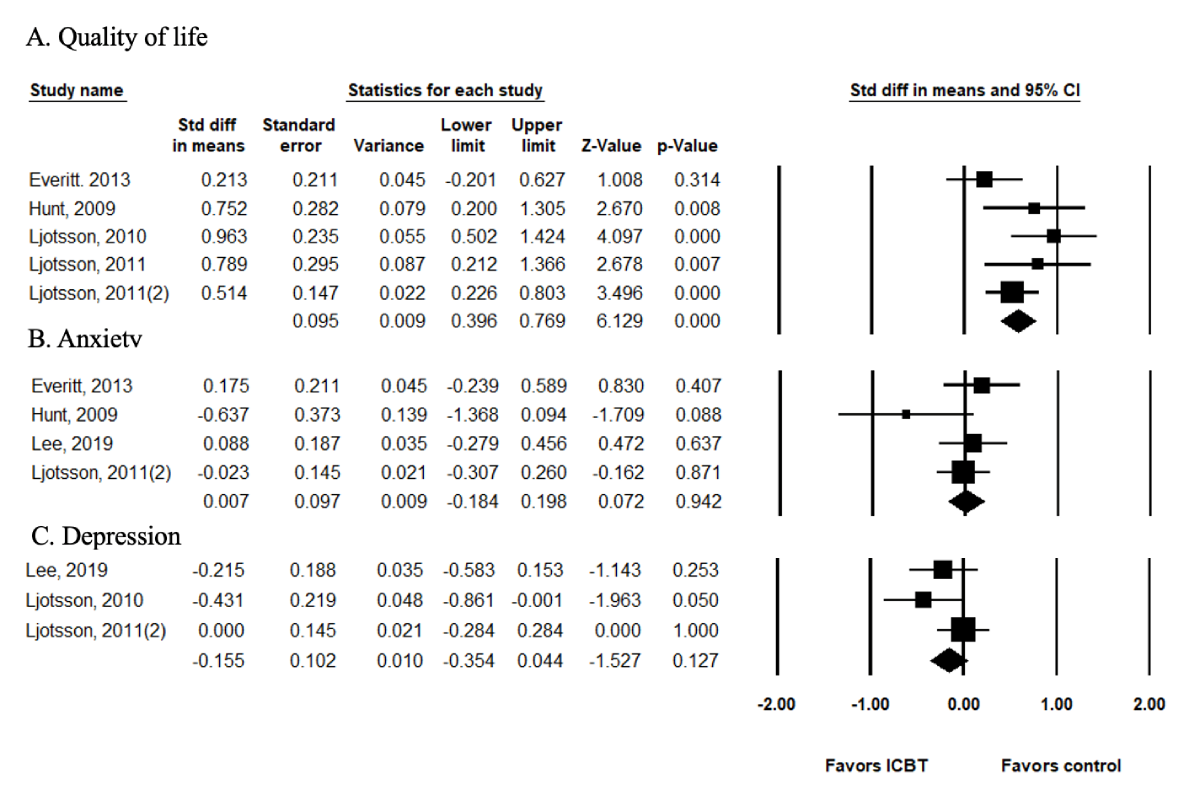
Effect size of ICBT on the quality of life and psychological status. ICBT: internet-delivered cognitive behavioral therapy.

Among the outcome variables, 5 studies [8,11,32-34] evaluated QOL using the same tool, the IBS-QOL developed by Patrick et al [20]. Therefore, the studies were not significantly heterogeneous (*I^2^*=40.71; *P*=.15), and the effect size was analyzed using a fixed model. The effect size of ICBT on the QOL of patients with IBS was significant at 0.582 (95% CI 0.396-0.769) compared with the control group (Figure 4A).

#### Psychological Status

To evaluate the effects of the ICBT on psychological status, the effect sizes on depression and anxiety were analyzed (Figure 4B and 4C). Psychological status was reported in 7 studies. However, 2 studies that integrated depression and anxiety were excluded from the meta-analysis [15,31], and 1 study was excluded from the analysis for depression because it did not provide an accurate mean score for depression [32]. Therefore, depression was analyzed in 3 studies [14,33,34] and anxiety in 4 [11,14,32,34], but neither was significantly heterogeneous (depression: *I^2^*=29.27 and *P*=.24; anxiety: *I^2^*= 22.11 and *P*=.28), so a fixed model was adopted. There was no evidence that ICBT had any effect on depression (SMD –0.155; 95% CI –0.354 to 0.044) or anxiety (SMD 0.007; 95% CI –0.184 to 0.198).

### Cost-Effectiveness

**Figure 5:**
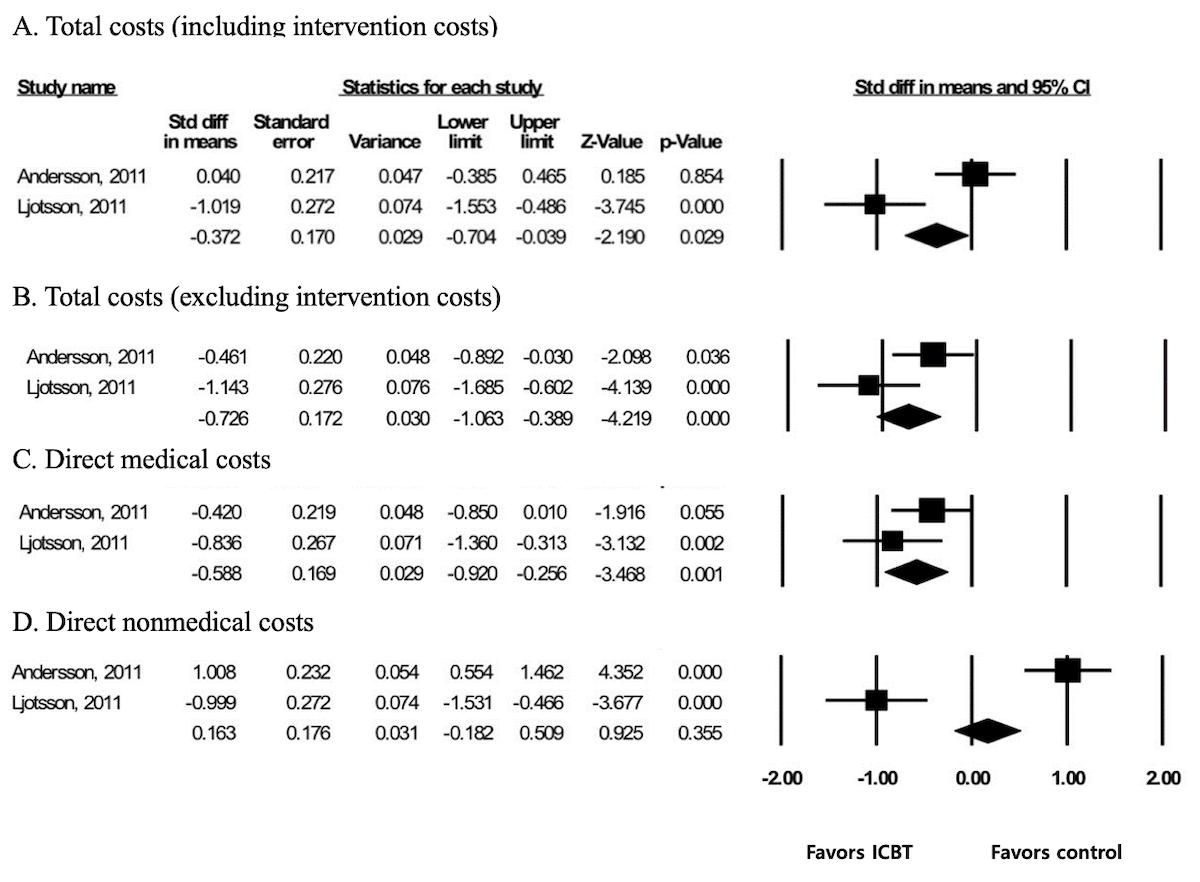
Effect size of ICBT on cost-effectiveness. ICBT: internet-delivered cognitive behavioral therapy.

Two studies [8,35] assessed the cost-effectiveness of ICBT. When analysis was performed with fixed models, there were significant reductions in total costs including intervention costs (SMD –0.372; 95% CI –0.704 to –0.039) and in total costs excluding intervention costs (SMD –0.726; 95% CI –1.063 to –0.389). In addition, a significant effect was found on direct medical costs (SMD –0.588; 95% CI –0.920 to –0.256), but no effect was found on the reduction of direct nonmedical costs (SMD 0.163; 95% CI –0.182 to 0.509; Figure 5).

### Publication Bias

Funnel plots could not be used in this study to evaluate publication bias because these plots require at least 10 studies. Instead, bias was evaluated using Egger’s regression intercept. The Egger intercepts were not significant in the analysis of outcome variables in this study, indicating that there was no risk for publication bias. Therefore, there was no need for a Duval and Tweedie’s trim and fill.

## Discussion

### Principal Results

To our knowledge, this is the first meta-analysis to evaluate ICBT as an effective treatment for patients with IBS. Nine RCT studies were included in the analysis, and their quality was generally acceptable. As these studies were heterogeneous due to the use of different intervention methods and measurement tools, it may be difficult to definitively determine the results of this meta-analysis.

In this study, ICBT showed an overall medium-to-large effect size during the postintervention evaluation in patients with IBS. Specifically, there were significant effects on IBS symptom severity, QOL, and cost-effectiveness. However, ICBT did not have an effect on the psychological status of the treatment group compared with the waiting list or standard care controls. When stratified by the type of ICBT intervention, both exposure therapy and self-management interventions were effective compared to controls. In the follow-up studies, the effects of ICBT on the severity of IBS symptoms remained. These findings are consistent with the results of a meta-analysis in which CBT was effective in treating IBS bowel symptoms and improving the QOL of patients with IBS [7]. Although the therapist’s contact is minimized in ICBT, our findings provide preliminary evidence that ICBT may be as effective as face-to-face CBT in patients with IBS.

Although only 2 RCTs among 9 studies reported the cost-effectiveness, the application of ICBT was found to improve clinical outcomes while reducing medical costs. Additional costs are required to provide ICBT, but the cost-reduction effect is maintained even after including the intervention costs. Furthermore, there was a significant effect on direct medical costs but not on nonmedical costs. Consistent with the McCrone et al [36] study, which evaluated CBT, there was no significant decrease in work days. Contrarily, in the treatment group, the improvement of IBS symptoms resulted in cost reduction compared with the control group [35]. This is consistent with our findings in the this study, in which ICBT showed significant effects on clinical outcomes.

Contrary to the results of this study, a recent CBT meta-analysis showed a significant improvement in psychological status [7]. However, in a recent review study of online psychological interventions in gastrointestinal disorders, a meta-analysis of 6 ICBT studies demonstrated no effect on stress, depression, anxiety, or QOL in patients with IBS [37]. This discrepancy may be because psychological status is not the primary outcome of ICBT. Unlike CBT with face-to-face intervention, ICBT with minimal therapist contact might not have significant effects on psychological status. Although ICBT is effective because it is not limited by time or location, having direct contact with therapists may provide additional benefits [38]. Support from therapists could also help participants improve their motivation and adherence to therapy, which would further enhance the effectiveness of ICBT [32]. In particular, for patients who suffer from more severe symptoms, direct contact with therapists could be beneficial. In future studies, it is necessary to evaluate the extent, content, and type of contact that would improve the effectiveness of the therapy. Moreover, even though ITT analysis was conducted, the levels of attrition were high in several of the RCTs we analyzed. In particular, the attrition rate ranged from 30% to 55% in studies with long-term follow-up [15,31]. This high attrition rate might be reduced with encouragement or motivation from therapists [11]. Refractory IBS, defined as persisting symptoms in a patient even after treatment for IBS is received, requires patients to continue to manage their symptoms [31], as ensuring the long-term effects of treatment is essential. Our findings demonstrated that the effect of ICBT on IBS symptom severity persisted for a period of 12 to 24 months after the final ICBT session. This showed that ICBT is a cost-effective intervention for IBS symptoms without the need for a booster session for a long period of time. However, for other variables in this study, the effects could not be analyzed because of the small number of RCTs. Therefore, more well-designed RCTs are required to verify the long-term effects of these outcome variables. Furthermore, it is necessary to determine how long after ICBT intervention a booster session would be required to sustain the effects.

### Limitations

This study has several limitations. First, there was a limited number of RCTs on ICBT for patients with IBS since research on ICBT only started recently. In particular, as 2 research teams conducted most of the ICBT trials on patients with IBS, there may be inherent biases in this meta-analysis. Our findings may be difficult to generalize to all IBS patients due to the low diversity in ethnicity and the similar characteristics of the participants. Certain limitations, such as Ljotsson’s team being unable to control for the expectancy of improvement by using a waiting list as a control group and Everitt’s team being unable to assess the treatment expectancy effects, indicate the importance of using an active control group. In addition, this meta-analysis may not reflect the effects of various ICBT programs or population groups. Therefore, our findings should be interpreted with caution, and further research on ICBT in different populations is needed. Second, some of the RCTs analyzed had small sample sizes, high attrition rates, and were heterogeneous, which may not substantially verify the effects of the interventions. Further research is warranted for RCTs through use of a large number of patients with IBS. Although a protocol was present for the ICBT used in each RCT, each protocol is different. Future studies should determine the effective content, frequency, and duration of ICBT.

### Conclusions

In conclusion, this meta-analysis demonstrated that ICBT was superior to standard care or being on a waiting list with regard to improving IBS symptom severity, QOL, and cost-effectiveness. The effects on IBS symptom severity persisted for a long time after the intervention; that is, ICBT can be considered an effective intervention that can be provided to patients with IBS regardless of location and time. However, the number of RCTs concerning the provision of ICBT to patients with IBS is still limited, and the protocols for ICBT, including content, duration, and operators, are heterogeneous, requiring further research and standardization. Nevertheless, this meta-analysis provides the first comprehensive insight into how ICBT could be used to improve the clinical outcomes and QOL of patients with IBS while reducing treatment costs.

## Appendix

Multimedia Appendix 1 Complete search strategy.

Multimedia Appendix 2 Summary of data extraction results.

## Abbreviations

CBT: cognitive behavioral therapy
CES-D: Center for Epidemiological Studies Depression scale
GSRS: Gastrointestinal Symptom Rating Scale
HADS: Hospital Anxiety and Depression Scale
IBS: irritable bowel syndrome
IBS-SSS: Irritable Bowel Syndrome Symptom Severity Scale
ICBT: internet-delivered cognitive behavioral therapy
ITT: intention to treat
KISS: Korean Studies Information Service System
KMBASE: Korean Medical Database
MADRS: Montgomery Asberg Depression Rating Scale
MSIT: Ministry of Science and ICT
PICO: Participant, Intervention, Comparator, Outcome
PRISMA: Preferred Reporting Items for Systematic Reviews and Meta-Analysis
QOL: quality of life
RCT: randomized controlled trial
RISS: Research Information Sharing Service
SMD: standardize mean difference
STAI-S: State-Trait Anxiety Inventory
TIC-P: Trimbos/Institute of Medical Technology Assessment Cost Questionnaire for Psychiatry
