# Supplementary material for: Internet-Delivered Cognitive Behavioral Therapy in Patients With Irritable Bowel Syndrome: Systematic Review and Meta-Analysis
Source: J Med Internet Res. 2022 Jun 10;24(6):e35260. doi: 10.2196/35260 (PMC9233255; doi:10.2196/35260)
Supplement: Multimedia Appendix 1 [file jmir_v24i6e35260_app1.docx]

**Appendix 1.** Complete search strategy

**PubMed**

- PubMed Advanced Search Builder
- Article type = clinical trial, randomized controlled trial
- Language – English, Korean

“irritable bowel syndrome”[Mesh] OR “irritable bowel syndromes” OR “syndrome, irritable bowel” OR “syndromes, irritable bowel” OR “irritable colon” OR “colon, irritable” OR “colitis, mucous” OR “colitidus, mucous” OR “mucous colitidus” OR “mucous colitis”

AND

“cognitive behavioral therapy”[Mesh] OR “cognitive behavioral therapies” OR “behavioral therapy, cognitive” OR “therapies, cognitive behavioral” OR “therapy, cognitive behavioral” OR “cognitive behavior therapy” OR “cognitive behavior therapies” OR “therapy, cognitive behavior” OR “therapies, cognitive behavior” OR OR “cognitive behaviour therapy” OR “cognitive behaviour therapies” OR “therapy, cognitive behaviour” OR “therapies, cognitive behaviour” OR “cognitive therapy” OR “cognitive therapies” OR “therapy, cognitive” OR “therapies, cognitive” OR “cognition therapy” OR “cognition therapies” OR “cognition therapies” OR “therapy, cognition” OR “therapies, cognition” OR “cognitive psychotherapy” OR “cognitive psychotherapies” OR “psychotherapy, cognitive” OR “psychotherapies, cognitive”

**Cochrane library**

- Advanced search
- Search manager = MeSH
- Search limit = content type: Trials

“Irritable bowel syndrome” OR “irritable bowel syndromes” OR “irritable colon”

AND

“cognitive behavioral therapy” OR “cognitive behavioral therapies” OR “cognitive behavior therapy” OR “cognitive behavior therapies” OR “cognitive therapy” OR “cognitive therapies” OR “cognitive behaviour therapy” OR “cognitive behaviour therapies” OR “cognition therapy” OR “cognition therapies” OR “cognitive psychotherapy” OR “cognitive psychotherapies”

**PsycINFO/KISS/KMBASE/RISS**

- Advanced search
- All Fields
- Document type = journal article
- Language = English, Korean

“Irritable bowel syndrome” OR “irritable bowel” OR “irritable colon”

AND

“cognitive behavioral therapy” OR “cognitive behavior therapy” OR “cognitive therapy” OR “cognitive behaviour therapy” OR “cognition therapy” OR “cognitive psychotherapy”

”
