# Supplementary material for: Internet-Delivered Cognitive Behavioral Therapy in Patients With Irritable Bowel Syndrome: Systematic Review and Meta-Analysis
Source: J Med Internet Res. 2022 Jun 10;24(6):e35260. doi: 10.2196/35260 (PMC9233255; doi:10.2196/35260)
Supplement: Multimedia Appendix 2 [file jmir_v24i6e35260_app2.docx]

**Appendix 2.** Summary of data extraction results.

| Authors | Primary outcome | Secondary outcome | Significant results | Nonsignificant results |
| --- | --- | --- | --- | --- |
| Andersson et al [35] | TIC-P^a^ | NA | ICER^b^: –16.806 Total cost: 16,988 vs 16,872 Indirect nonmedical cost: 11,227 vs 12,619 | Direct medical costs: 3222 vs 3547 Direct nonmedical cost: 958 vs 571 |
| Everitt et al [32] | 1) IBS-SSS^c^ 2) IBS-QOL^d^ | 3) PEQ^e^ 4) SGA^f^ relief 5) HADS-A^g^ 6) HADS-D^h^ | 1) 197.0 vs 162.8 3) 6-12 score: 28.9% vs 13.5% | 2) 71.6 vs 69.4 4) 6-12 score: 35.5 vs 25.6 5) 8.7 vs 9.2 |
| Everitt et al [15] | 1) IBS-SSS 2) WSAS^i^ | 3) HADS 4) PEQ 5) SGA relief | 1) 3 m^j^: 179.1 vs 236.5 6 m: 170.3 vs 212.7 12 m: 163.0 vs 205.6 2) 3 m: 9.4 vs 12.1 6 m: 7.6 vs 10.5 12 m: 7.4 vs 10.8 3) 3 m: 13.4 vs 15.6 6 m: 12.7 vs 15.2 12 m: 12.7 vs 15.0 4) 3 m: 6.6; 6 m: 4.8; 12 m: 3.5 5) 3 m: 6.4; 6 m: 5.0; 12 m: 3.6 | N/A^k^ |
| Everitt et al [31] | 1) IBS-SSS 2) WSAS | 3) HADS 4) PEQ | 2) 7.3 vs 9.7 3) 12.2 vs 15.1 4) 3.3 | 1) 167.6 vs 197.9 |
| Hunt et al [11] | 1) GSRS-IBS^l^ | 2) IBS-QOL 3) ASI-GI^m^ 4) CPSQ-Gi^n^ | 1) 35 vs 52 2) 84 vs 111 3) 1.9 s 2.5 4) 1.25 vs 2.1 | N/A |
| Lee et al  [14] | 1) BSSS^o^ | 2) STAI-S^p^ 3) CES-D^q^ | Estimated mean 1) 6 w^r^: –2.24 vs –6.72 2) 18 w: –14.54 vs –8.40 3) 2 w: –2.28 vs 1.68 18 w: –9.15 vs –3.44 | 1) 2 w: –4.66 vs –3.00 18 w: –9.90 vs –7.04 2) 2 w: –2.26 vs –1.08 6 w: –4.61 vs –5.45 3) 6 w: –3.28 vs –1.40 |
| Ljótsson et al [33] | 1) GI^s^ symptom diary 2) GSRS-IBS | 3) IBS-QOL 4) VSI^t^ 5) MADRS-S^u^ | 1) Total pain: 1.4 vs 2.4 Diarrhea: 0.4 vs 0.6 Blotating: 0.9 vs 1.7 Nausea: 0.6 vs 0.6 Flatulence: 0.9 vs 1.4 2) 32.4 vs 47.3 3) 72.8 vs 52.9 4) 30.2 vs 41.9 5) 6.9 vs 10.5 | 1) Constipation: 0.3 vs 0.7 Belching: 0.4 vs 0.5 |
| Ljótsson et al [8] | 1) GSRS-IBS | 2) TIC-P 3) IBS-QOL 4) VSI | 1) 31.0 vs 40.9 2) ICER: –39.821  total costs: 15,014 vs 18,323 Direct medical costs: 1148 vs 1453 3) 82.6 vs 67.4 4) 14.1 vs 26.2 | 2) Direct nonmedical cost: 150 vs 338 Indirect nonmedical costs: 12729 vs 16233 |
| Ljótsson et al [34] | (1) GSRS-IBS | (2) IBS-QOL (3) VSI (4) CSFBD^v^ (5) PSS^w^ (6) HADS-A (7) HADS-D (8) SGA relief | (1) Post: 36.3 vs 41.1  6 m: 33.4 vs 39.3 (2) Post: 75.7 vs 65.7 6 m: 74.9 vs 68.7 (3) Post: 24.9 vs 30.5 6 m: 23.1 vs 29.4 (4) Post: 100.3 vs 120.7 6 m: 101.6 vs 116.0 (8) 6 m: 65% vs 44% | (5) Post: 15.0 vs 14.8, 6 m: 15.5 vs 16.0 (6) Post: 7.2 vs 7.3, 6 m: 7.2 vs 7.8 (7) Post: 4.4 vs 4.4 6m: 4.4 vs 4.7 (8) Post: 69% vs 58% |

^a^TIC-P: Trimbos/iMTA questionnaire for costs associated with psychiatric illness.

^b^ICER: incremental cost-effectiveness ratio.

^c^IBS-SSS: Irritable Bowel Syndrome Symptom Severity Scale.

^d^IBS-QOL: Irritable Bowel Syndrome Quality of Life Scale.

^e^PEQ: patient enablement questionnaire.

^f^SGA: subject’s global assessment.

^g^HADS-A: Hospital Anxiety and Depression Scale-Anxiety.

^h^HADS-D: Hospital Anxiety and Depression Scale-Depression.

^i^WSAS: Work and Social Adjustment Scale.

^j^m: month.

^k^N/A: not applicable.

^l^GSRS: Gastrointestinal Symptom Rating Scale.

^m^ASI-GI: Anxiety Sensitivity Index-Gastrointestinal.

^n^CPSQ-GI, Consequences of Physical Sensations Questionnaire-Gastrointestinal.

^o^BSSS: Bowel Symptom Severity Scale.

^p^STAI-S: State-Trait Anxiety Inventory.

^q^CES-D: Center for Epidemiological Studies Depression Scale.

^r^w: weeks.

^s^GI: gastrointestinal.

^t^VSI: Visceral Sensitivity Index.

^u^MADRS-S: Montgomery Asberg Depression Rating Scale.

^v^CSFBD: Cognitive Scale for Functional Bowel Disorders.

^w^PSS: Perceived Stress Scale.
